# A Coherent Assessment of the Compressive Strain Rate Response of PC, PETG, PMMA, and TPU Thermoplastics in MEX Additive Manufacturing

**DOI:** 10.3390/polym15193926

**Published:** 2023-09-28

**Authors:** Markos Petousis, Ioannis Ntintakis, Constantine David, Dimitrios Sagris, Nektarios K. Nasikas, Apostolos Korlos, Amalia Moutsopoulou, Nectarios Vidakis

**Affiliations:** 1 Department of Mechanical Engineering, Hellenic Mediterranean University, 71410 Heraklion, Greece; markospetousis@hmu.gr (M.P.); ntintakis@hmu.gr (I.N.); amalia@hmu.gr (A.M.); 2 Department of Mechanical Engineering, International Hellenic University, Serres Campus, 62124 Serres, Greece; david@ihu.gr (C.D.); dsagris@ihu.gr (D.S.); 3 Division of Mathematics and Engineering Sciences, Department of Military Sciences, Hellenic Army Academy, 16673 Vari, Greece; nasikas@sse.gr; 4 Department of Industrial Engineering and Management, International Hellenic University, 14th km Thessaloniki—N. Moudania, Thermi, 57001 Thessaloniki, Greece; apkorlos@ihu.gr

**Keywords:** 3D printing, fused filament fabrication, additive manufacturing, compression strain sensitivity index, compression test, thermoplastics, material extrusion

## Abstract

In this study, we successfully address a significant research and engineering gap by quantitatively assessing the impact of varying compressive loading rates on the mechanical behavior of four popular thermoplastic polymers in material-extrusion-based (MEX) 3D printing. Raw powders of polycarbonate (PC), polyethylene terephthalate glycol (PETG), polymethyl methacrylate (PMMA), and thermoplastic polyurethane (TPU) were processed through melt extrusion, and the filaments were used to 3D-print the test samples. For completeness, thermogravimetric analysis and a compressive test following the ASTM-D695 standard were conducted. Ultimately, the compressive strength and yield stress, the compressive modulus of elasticity and toughness, and the maximum compressive sensitivity index were thoroughly documented. Specimens were tested in strain rates from 1.3 mm/min to 200 mm/min. The compressive strength (40% for the PMMA) and stiffness (29% for the TPU) increased with the increase in the strain rate in all polymers tested. PC had the highest strain rate sensitivity. Significant variations in deformation and fracture modes were observed and thoroughly documented throughout this study. Our findings can be useful in industrial engineering as valued design optimization input parameters in various applications involving the above-mentioned polymeric materials.

## 1. Introduction

Recent advances in additive manufacturing have created great prospects for both researchers and the industry, as they have opened up a huge spectrum of potential applications and processes [1]. Indisputably, additive manufacturing (also widely termed 3D printing) has started a new era for the manufacturing industry, as has been evident over the last few decades [2]. Transitioning from rapid prototyping to additive manufacturing, new technological advances have been achieved, and new milestones have been reached [3]. Novel materials that concern industrial production have been developed that have allowed for novel functionalities and processes to be realized [4,5,6]. One of the most significant advantages of additive manufacturing is the fabrication of complex structures from a variety of materials and their subsequent use in novel applications involving, in most cases, extreme environments and high demands for their performance [7,8,9]. Thus, it is important to investigate the mechanical behavior of various materials under various conditions, such as their mechanical response under loading stress and strain, etc. [10,11].

One of the most popular and well-developed categories of materials for additive manufacturing is thermoplastics, which are widely used in the fused filament fabrication (FFF) technique [12,13,14]. Beyond common thermoplastic materials such as polylactic acid (PLA) [15,16,17] and acrylonitrile butadiene styrene (ABS) [18,19], novel materials have started to be used in additive manufacturing recently. Among them are widely used polymeric materials such as PC (polycarbonate) [20,21], TPU (thermoplastic polyurethane) [22,23], PETG (polyethylene terephthalate glycol) [24,25], and PMMA (polymethyl methacrylate) [26,27,28].

The main mechanical property of the PC thermoplastics is their resistance to fracture and impact [29]. PC materials are considered environmentally friendly materials which are suitable for 3D printing [20]. Owing to its chemical characteristics, PC demonstrates very good chemical resistance [30,31], which is very important for relevant applications. Also, PETG exhibits good chemical resistance and is characterized by significant durability and formability [32,33]. PETG, compared to PET, exhibits superior impact resistance, strength, and durability [32]. Its low heat-forming temperature and excellent layer adhesion make PETG a widely used material for additive manufacturing, for instance in large prints, thanks to its low shrinkage rates [34,35,36]. PMMA stands out for its great mechanical performance and can be successfully 3D-printed in various shapes and forms [37,38]. Thermoplastic polyurethane (TPU) is an abrasion-resistant and flexible material which encompasses the characteristics of both plastic and rubber [39]. Mechanical properties such as high flexibility, high tensile strength, and increased durability usually characterize TPU-fabricated 3D printed parts. Moreover, TPU-printed structures can withstand high ambient temperatures [40], which makes them ideal materials for relevant applications where increased temperatures are involved.

In several papers in the literature, FFF-processed polymers have been studied in the past under various loading conditions [41,42]. Studies have also focused on the strain rate sensitivity of AM-processed polymeric materials [43,44,45,46]. This leaves plenty of room for the study of the behavior of polymeric AM materials on compressive loading, which has not been extensively studied so far [47]. The importance of compressive loading in mechanical structures has been frequently reported [48,49]. These types of loadings, particularly when applied to 3D components, are not frequently examined in the academic literature. This scarcity of research may be attributed to the substantial sample sizes needed for testing, which demand considerable material resources and time in order to align with the relevant testing standards [50].

Our current research focuses on elucidating the performance under various strain rates in the compression loading of four different polymeric materials which are popular for different types of applications, i.e., PC, PETG, PMMA, and TPU, respectively, produced for AM. The four polymers mentioned above were prepared using a thermomechanical extrusion process from raw materials. The produced samples, after they were 3D-printed, were tested with five different strain rates and speeds of testing (up to 200 mm/min). Varying the strain rate has been found to highly affect the performance of polymeric materials [51]. To the authors’ best knowledge, no similar research has been present so far in the literature. The provided information herein is crucial since the strain rate and compression sensitivity have been used as design factors [52] in developing fail-safe systems and evaluating rate-dependent responses such as energy absorption during the failure of parts and similar scenarios [53]. The index of strain rate sensitivity, also known as the “*m*” index, is derived from the following Equation (1) as a function of the strain (%) [46,54]:

(1) $$m=\frac{\Delta ln\left(\sigma \right)}{\Delta ln(\dot{\varepsilon )}}$$

The effect of the strain rate under compressive loading in these four popular polymeric materials in FFF 3D printing is reported herein for the first time, thus providing valuable information for the mechanical performance and behavior of these materials when 3D-printed and subjected to such types of loading. Detailed knowledge of the performance of these materials under harsh conditions is extremely important in the defense and security sector as well, where there is a constant need for novel materials with enhanced performance and functionalities.

## 2. Materials and Methods

### 2.1. Raw Thermoplastics for Melt Extrusion

Initially, the raw thermoplastics were procured in a pellet form. PC was procured from Styron (granules, density of 1200 g/cm^3^, melt flow index of 15 g/10 min, Samstagern, Switzerland), PETG from Felfil (pellets, Turin, Italy), PMMA (pellets, 1183 g/cm^3^, melt flow index of 1133 mL/10 min) from JULIER (Fujian, China), and TPU from Ravago PetrokimyaSatisVE (pellets, density of 1160 g/cm^3^, Istanbul, Turkey).

### 2.2. Methods

**Figure 1:**
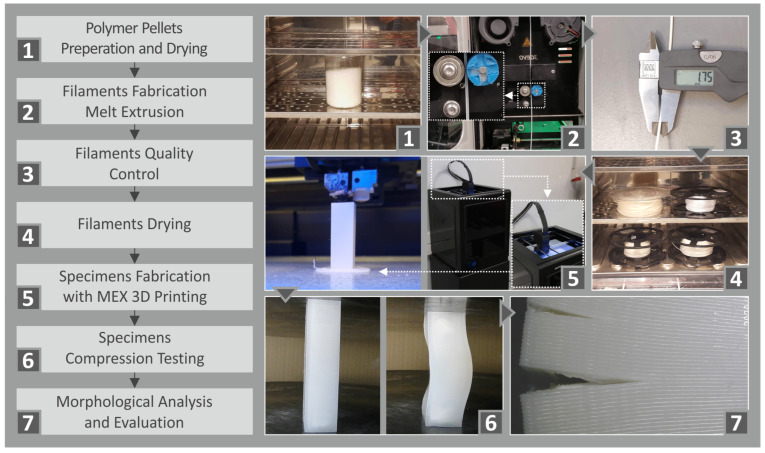
The flow chart of the adopted methodology presented on the left side of the figure involves the individual phases from the preparation of the specimens, the extrusion process, and the fabricated phase to, finally, the testing procedure. On the right side, pictures from the stages of the experimental procedure followed are presented.

The flow chart diagram Figure 1 depicts the adopted methodology from the raw materials’ preparation until the compression test’s execution. All stages are analytically presented in the following sections.

#### 2.2.1. Filament Preparation and Extrusion

The preparation of the filament started with the raw materials drying under fully controlled conditions to remove any absorbed moisture (50 °C, 10–12 h). After that, the filament extrusion stage followed, employing the 3D Evo Composer 450 desktop extruder (3D Evo). The nominal diameter of the filament was 1.75 mm with a tolerance of 0.07 mm. The filament diameter was controlled continuously through a built-in optical sensor. The raw material came through four different heating zones during the extrusion process in the chamber of the extruder. The number 1 heating zone was close to the nozzle, whereas heating zone number 4 was next to the crucible. The temperature of each heating zone had to be controlled, depending on the polymer and its properties. Other parameters under consideration were the rotational speed of the cooling fans, the winder, and the screw of the extruder. Table 1 shows the extrusion specifications for each polymer material. After the extrusion process, the diameter and the roughness of the filaments were checked using appropriate manual instrumentation.

#### 2.2.2. Fabrication of the Compression-Testing Specimens

**Figure 2:**
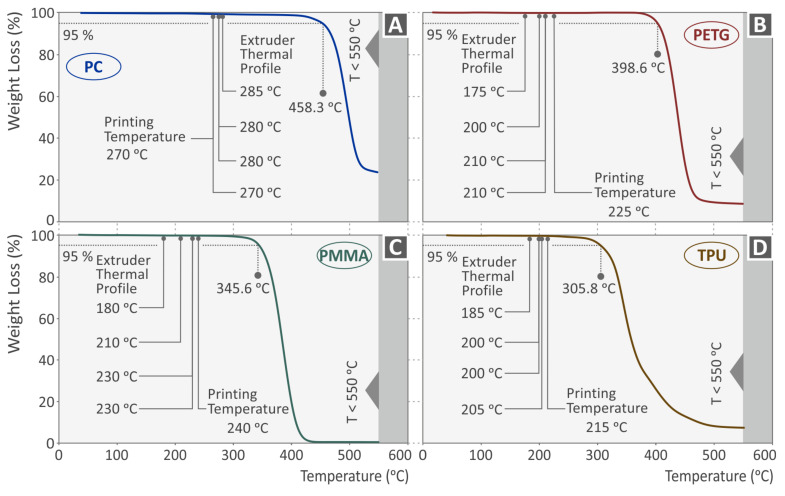
Investigation with TGA of the thermal properties for each polymer studied (**A**) PC, (**B**) PETG, (**C**) PMMA, (**D**) TPU.

The AM specimens were manufactured in accordance with the American Society for Testing and Materials (ASTM) D695-02a standard [55]. For each material and test speed, five specimens were 3D-printed by means of the FFF technique (Intamsys Funmat HT 3D printer). Before initiating the 3D-printing process, the filaments were oven-dried to remove any further moisture for four hours at 50 °C. The MEX process settings for all specimens were the same regarding the infill (100%) and the shell pattern. The nozzle used had a diameter of 0.4 mm. In order to determine the 3D printing settings for each polymer, a preparatory experimental process was followed. Additionally, we employed thermogravimetric analysis (TGA) and revealed the thermal properties of each material. A PerkinElmer Diamond apparatus (PerkinElmer, Inc., Waltham, MA, USA) was used. Measurements were conducted in a nitrogen environment at a rate of 10 °C/minute and a temperature range of 25–550 °C. The initial weight of the sample was approximately 8 mg. Correlating the outcome of the TGA with the applied temperatures ensured that the extrusion temperatures used were not affecting the polymers’ thermal stability (Figure 2). Table 2 presents the various additive manufacturing settings for each polymer.

#### 2.2.3. Testing Procedure for the Compression of the Materials

The Instron KN1200 (Norwood, MA, USA) universal material testing machine was used for the conduction of the compression tests. To evaluate the behavior of each polymer under different conditions, the compression speed was varied from 1.3 (as per the ASTM D695) to 200 mm/min. In particular, the selected testing speeds were 1.3, 50, 100, 150, and 200 mm/min to cover a broad range of compression loading rates representative of potential working conditions. The work aimed to evaluate the mechanical performance of the specific polymers at high test speeds, especially since the standard (ASTM D695) instructs using a low test speed on the samples. For completeness, to be consistent with the standard and to be able to compare the results with the ones provided with the standard’s conditions, the test speed of the standard (1.3 mm/min) was also tested. For each case (polymer and strain rate), five specimens were tested at a room temperature of 23 °C.

## 3. Results

### 3.1. Compression Test Results

**Figure 3:**
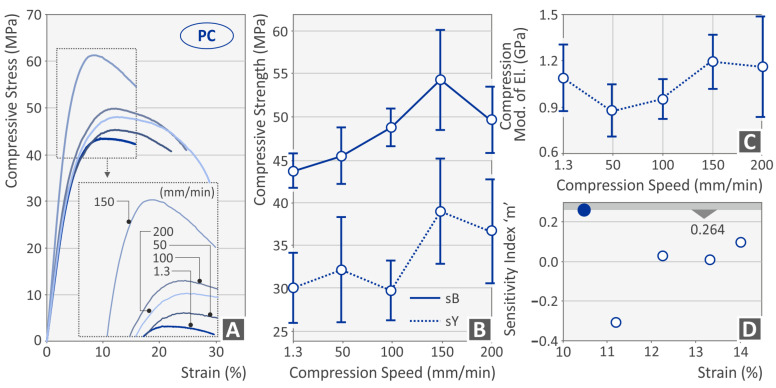
PC polymer (**A**) compression stress–strain (MPa-%) curve at various compression speeds (the graph of one randomly selected sample of the five tested from the specific polymer at each speed is presented), (**B**) compression strength, (**C**) compressive modulus, and (**D**) index m—strain (%) graph (blue dot indicates the highest sensitivity index m recorded for the PC polymer).

**Figure 4:**
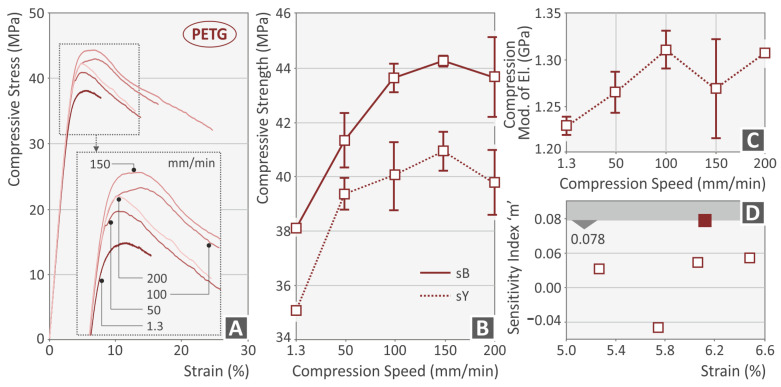
PETG polymer (**A**) compression stress–strain (MPa-%) curve at various compression speeds (the graph of one randomly selected sample of the five tested from the specific polymer at each speed is presented), (**B**) compression strength, (**C**) compressive modulus, and (**D**) index m—strain (%) graph (burgundy square indicates the highest sensitivity index m recorded for the PETG polymer).

**Figure 5:**
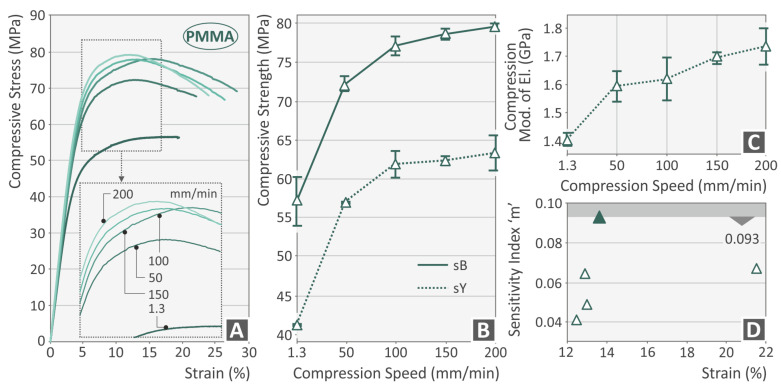
PMMA polymer (**A**) compression stress–strain (MPa-%) curve at various compression speeds (the graph of one randomly selected sample of the five tested from the specific polymer at each speed is presented), (**B**) compression strength, (**C**) compressive modulus, and (**D**) index m—strain (%) graph graph (green triangle indicates the highest sensitivity index m recorded for the PMMA polymer).

**Figure 6:**
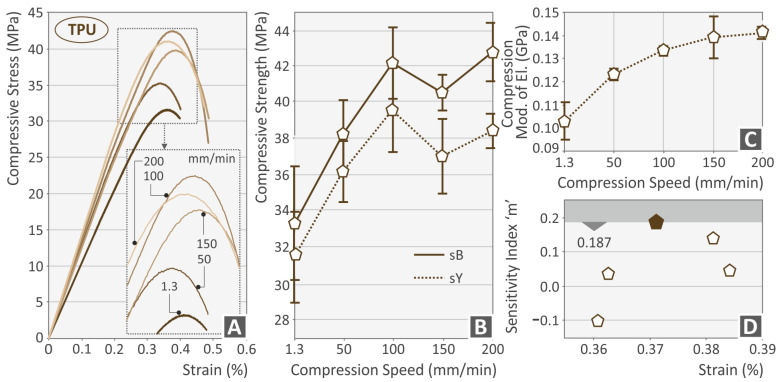
TPU polymer (**A**) compression stress–strain (MPa-%) curve at various compression speeds (the graph of one randomly selected sample of the five tested from the specific polymer at each speed is presented), (**B**) compression strength, (**C**) compressive modulus, and (**D**) index m—strain (%) graph graph (brown pentagon indicates the highest sensitivity index m recorded for the TPU polymer).

Figure 3, Figure 4, Figure 5 and Figure 6 present the compression test results for the four thermoplastics tested. In general, any rise of the strain rate subsequently increases the achieved strength, up to a specific value, which agrees well with the literature [56]. For the PC (Figure 3B) and the PETG (Figure 4B) polymer, the highest strength was achieved at 150 mm/min speed of testing, while for the PMMA (Figure 5B) and the TPU (Figure 6B) materials, this was achieved at the 200 mm/min speed, respectively. The corresponding yield stress seems to follow a similar pattern for all four polymers. The increase in the strain rate makes the PC (Figure 3A) and the PETG (Figure 4A) polymers more ductile while having no significant effect on the other two polymeric materials (PMMA, Figure 5A; TPU, Figure 6A). On the other hand, the compression modulus of elasticity does not seem to follow the same pattern for the polycarbonate (Figure 3C) and the PETG (Figure 3C) polymers. The highest values were found at 150 mm/min and 100 mm/min speeds of testing, respectively. For the PMMA (Figure 5C) and the TPU (Figure 6C) polymers, the compression modulus seems to follow the same pattern as the strength values. Regarding the sensitivity index, lower strain rate values seem to increase it for the PC (Figure 3D) and the PMMA (Figure 5D) polymers. In the PETG, it seems to have the opposite effect (Figure 4D), while for the TPU polymer (Figure 6D), the median values are found to exhibit the highest strain sensitivity. The deviation between the results was higher for the PC (Figure 3B) and the TPU (Figure 6B) polymers, while the PETG (Figure 4B) and the PMMA (Figure 5B) thermoplastics showed very low deviations in their results between the various samples.

**Figure 7:**
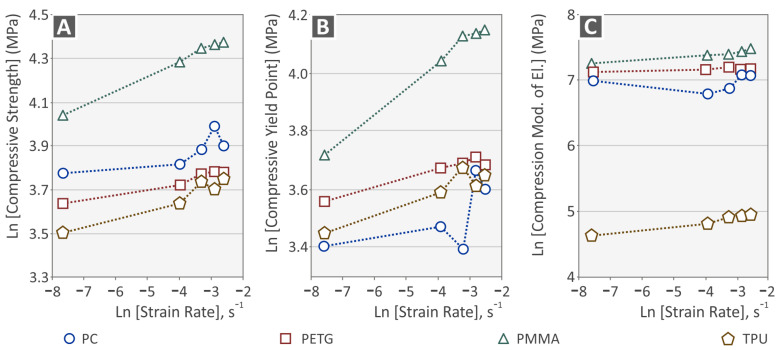
(**A**) Compressive strength vs. strain rate, (**B**) compressive yield point (MPa), and (**C**) compression modulus of elasticity (MPa).

Figure 7 demonstrates comparative graphs in logarithmic scale between the four examined polymers for their compression properties under various compressive loading rates. Figure 7A shows the maximum compressive strength, whereas Figure 7B demonstrates the maximum compressive yield point, and Figure 7C the compressive modulus of elasticity for the various strain rates (s^−1^). The PMMA polymer shows a more intense increase in its properties compared to the other polymers and has, overall, the highest response under compression loads. The other three polymers show a similar trend regarding their compressive strength, suggesting similar mechanisms governing these properties. The PC polymer differs in its response to yield strength and the compression modulus.

**Figure 8:**
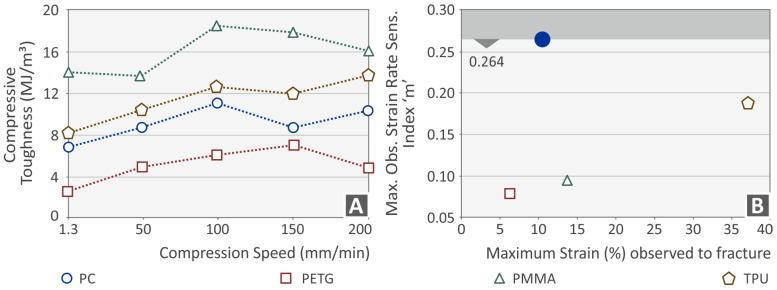
(**A**) Compressive toughness vs. compression speed and (**B**) maximum determined index m vs. the maximum strain observed (blue dot indicates the highest sensitivity index m recorded for the PC polymer, the other shapes and colors refer to the remaining materials tested, according to the provided legend in the figure).

The calculated toughness (absorbed energy during the tests, calculated as the integral of the stress–strain curve) of each polymer for all compression speeds is shown in Figure 8A. From the results, it is obvious that the toughness of PMMA is much higher than that of the three other polymers, while PETG exhibits the lowest toughness among the four polymers tested. The PC and TPU polymers seem to have a rather similar value of compressive toughness. Figure 8B presents the determined compression index “m” vs. the maximum strain (%) observed at fracture. The PC polymer seems to be highly dependent on the strain rate. Conversely, the PETG and PMMA polymers have lower dependences on the strain rate effect (“m”).

### 3.2. Morphological Characteristics of the Compression Specimens

**Figure 9:**
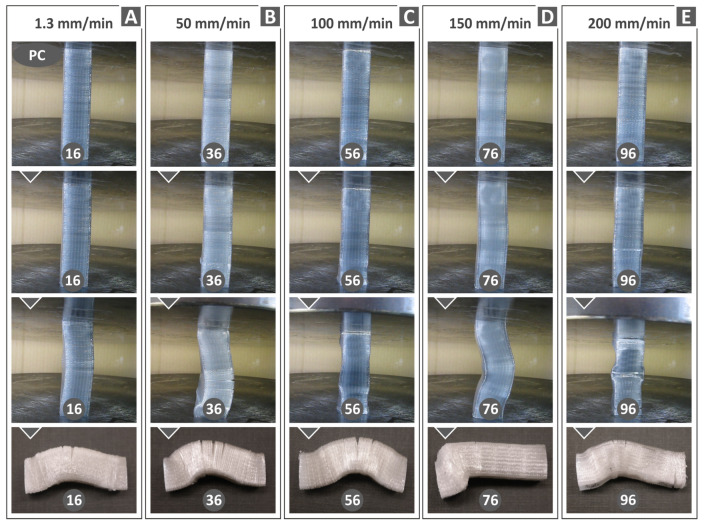
PC polymer specimens’ deformation at different compression speeds: (**A**) 1.3 mm/min, (**B**) 50 mm/min, (**C**) 100 mm/min, (**D**) 150 mm/min, and (**E**) 200 mm/min, respectively.

According to the performed compression tests of the examined polymers, it seems that the specimens, when reaching the ultimate compressive stress, failed in either of two modes, namely flexural buckling or flexural buckling associated with out-of-plane kinking. The second failure mode is the most common at high strain rates. Figure 9 refers to the PC polymer failure under axial compressive loading for various compression speeds. For a 1.3 mm/min compression speed, some cracks are restricted at the maximum deflection area in the lateral direction as a result of buckling (Figure 9A). Upon increasing the compression speed to 50 mm/min, the cracks grow further (Figure 9B). Figure 9C shows the specimens’ compressive failure for a 100 mm/min compression speed. It is evident that the damage in the cracked area is reduced. By further increasing the strain rate, the polymer structure is subjected to a yielding mechanism, thus resulting in a limited fracture (Figure 9C–E).

**Figure 10:**
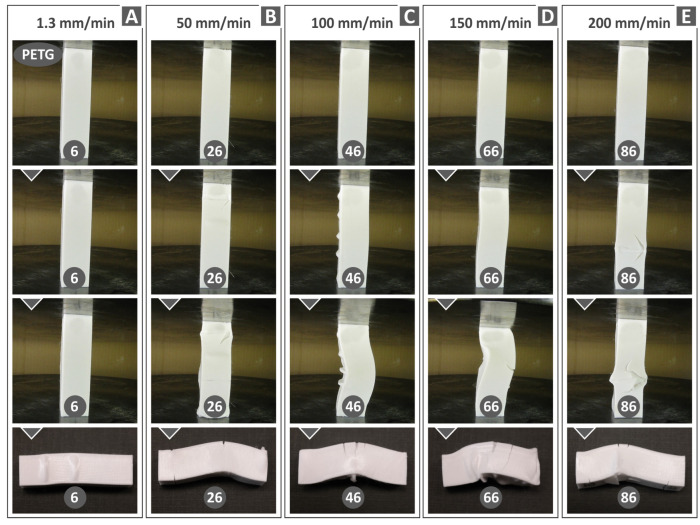
PETG polymer specimens’ deformation at different compression speeds: (**A**) 1.3 mm/min, (**B**) 50 mm/min, (**C**) 100 mm/min, (**D**) 150 mm/min, and (**E**) 200 mm/min.

**Figure 11:**
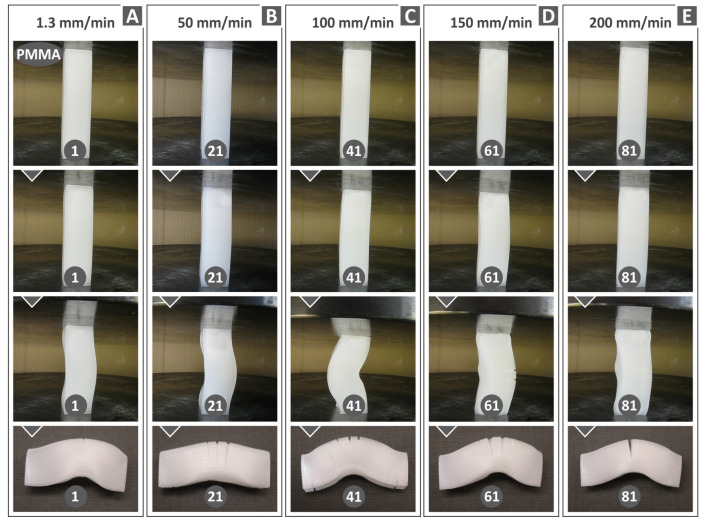
PMMA polymer specimens’ deformation at different compression speeds: (**A**) 1.3 mm/min, (**B**) 50 mm/min, (**C**) 100 mm/min, (**D**) 150 mm/min, and (**E**) 200 mm/min.

**Figure 12:**
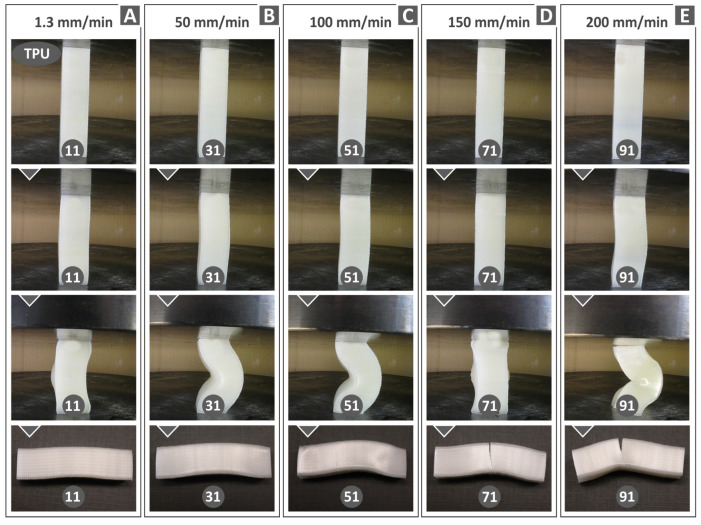
TPU polymer specimens’ deformation at different compression speeds: (**A**) 1.3 mm/min, (**B**) 50 mm/min, (**C**) 100 mm/min, (**D**) 150 mm/min, and (**E**) 200 mm/min.

Figure 10, Figure 11 and Figure 12 display compression test experiment images at varying strain rates for the remaining materials. Notable distinctions can be observed in the way the samples respond both in terms of their deformation and the development of cracks upon failure. For instance, when subjected to different test speeds, the PC and TPU samples adopt an “S”-shaped deformation, whereas the PETG and PMMA samples exhibit a “C”-shaped deformation. Furthermore, all polymers except TPU consistently exhibit cracks across all test speeds. The TPU polymer samples primarily display significant cracks at the highest speeds tested (150 and 200 mm/min, as seen in Figure 10C).

**Figure 13:**
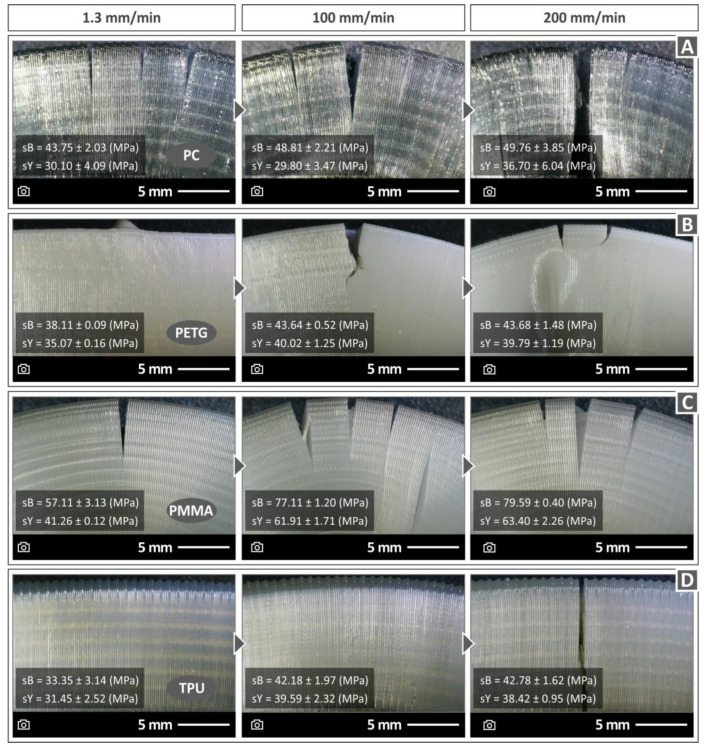
Morphological characteristics at 1.3, 100, and 200 mm/min compression speeds for (**A**) PC, (**B**) PETG, (**C**) PMMA, and (**D**) TPU polymer samples.

The tested specimens were also evaluated regarding their morphological characteristics after being subjected to compression testing by means of optical microscopy (Kern OKO 1). Figure 13 summarizes the results regarding the compressive performance of all four polymers examined in this work. The morphological characteristics of the specimens that failed under compressive loads are illustrated for three indicative compression speeds (i.e., 1.3, 100, and 200 mm/min). The results show that the PC specimen tested at 1.3 mm/min failed to exhibit multiple cracks, in some cases even at the outer surface of the 3D-printed part, where the tensile stress intensity due to the buckling deflection is mostly accommodated (Figure 13A). At the 100 mm/min strain rate, extensive cracking was observed (Figure 13A). The PC specimen was completely fractured at a 200 mm/min compression speed (Figure 13A). Figure 13B shows that, for the PETG specimen at 1.3 and 100 mm/min rates, there is no fracture damage in the 3D-printed polymer structure. PETG was fractured at a 100 mm/min compression speed (Figure 13B). At a 200 mm/min compression speed, cracks and wrinkles were observed on the structure of the PETG specimen (Figure 13B). Figure 13C shows the compressive fracture mode of the PMMA specimens. It was observed that cracking takes place at a compression speed of 1.3 mm/min with a tendency to become larger for 100 and 200 mm/min compression speeds. The TPU test specimen (Figure 13D), compared to the other polymers examined, presents an outstanding deformation without any cracking failure at most of the range of the strain rates (1.3 and 100 mm/min compression speeds). A 90-degree crack is shown only at the highest strain rate of 200 mm/min. In the image, the entire specimen is not shown. This is a zoomed-in picture of the crack. TPU is not a brittle material. This crack was probably due to the layers’ fusion collapsing at this test speed, as these are 3D-printed samples, and the 3D printing structure affects their mechanical performance.

## 4. Discussion

Polymers display viscoelastic characteristics; thus, their mechanical behavior is significantly influenced by both the speed of application of an external mechanical force and the temperature of the surrounding environment [57]. Additionally, the strain hardening effect (i.e., as the strain increases, the materials become more stiff and show increased strength [58]) was verified herein. As the strain rate applied during the experimental procedure increased, the compressive strength of all four polymers also increased. The temperature remained constant throughout these experiments, and its effect was not within the scope of the study. At low strain rates, strain hardening was not anticipated. The strengthening mechanism can be potentially attributed to some level of alignment or orientation of the polymeric chains, which consequently enhanced the polymers’ toughness [57]. Furthermore, the four types of polymers exhibited varying degrees of strain hardening in the experimental procedure, which was the expected outcome [57]. The PC polymer had a significantly higher strain sensitivity index “m” than the other three polymers. Additionally, the PC polymer showed a decrease in the compression strength at the highest speed tested. This decrease was probably due to the fact that there is a limit to the positive effect of strain hardening on the mechanical properties of each polymer. Such a behavior is consistent with the corresponding literature [51,59]. The TPU had the second-highest strain sensitivity index “m,” while the other two polymers had similar and much lower values. Although polymers are frequently employed in applications involving load engineering loads, it is crucial to emphasize the significance of parameters such as the strain sensitivity index “m” during material selection, as the application of engineering loads with high strain rates can potentially result in a “catastrophic fracture” of the structure. This is even more important in 3D-printed parts which exhibit anisotropic behavior, affecting their mechanical performance. This behavior is also affected by the fusion and the bonding between the strands and the layers of the 3D printing structure [60].

The rationale for emphasizing the significance of compressive loadings and the investigation of polymeric materials has been substantiated and documented in the research. The variations observed in the response of the four polymeric materials further validate the importance of such research endeavors, particularly in the context of 3D-printed components. This research also encompasses an evaluation of how the manufacturing process affects the performance of the resulting components. The findings can be harnessed in the design of these components. They exhibit the capacity to endure higher engineering loads, particularly in situations where operational conditions entail higher strain rates for the applied engineering loads. Notably, there is a significant enhancement in compressive strength with increases of 20% for the PC polymer, 15% for the PETG, a vast 40% for the PMMA polymer, and 23% for the TPU polymer. The yield stress demonstrated a similar elevation and response to the compressive strength across all four polymers.

Moreover, the modulus of elasticity undergoes elevation in three of the four polymers tested. Only the modulus of elasticity of the PC polymer decreased with the increase in the strain rate. The increase in the modulus of elasticity signifies that, with higher strain rates, the polymers demonstrate greater rigidity, resulting in decreased deformation under equivalent engineering loads. The increase in the modulus of elasticity reached 8% for the PETG polymer, 18% for the PMMA, and a vast 29% for the TPU polymer. As a result, this research empowers the design of lighter-weight structures, the augmentation of the safety margins, and the development of stiffer components with reduced dimensions when the engineering loading scenarios for a structure include such types of loadings.

The literature review showed that polymers have been studied for their behavior with high strain rates [61,62], but concerning compressive loadings, the literature is still limited. The PC polymer [63,64], PETG [65], and PMMA [66,67,68] have been investigated in bulk form. The TPU polymer has been studied in both bulk [69] and 3D-printed forms [70,71]. Although the results presented herein cannot be directly correlated with these studies due to the different forms of the materials and different ranges in the applied engineering loads rate, it can be safely concluded that they align closely with the previous research. Additionally, aspects such as compression toughness and the strain rate sensitivity index were not extensively assessed in most of these previous works. In light of these considerations, the evaluation of the existing literature showed that the findings herein are reliable. The lack of similar investigations so far for these polymers also supports the merit of the presented findings. As noted above, the four polymers studied have unique properties that make them suitable for specific types of applications in which the most popular polymers in 3D printing, such as acrylonitrile butadiene styrene (ABS) and polylactic acid (PLA), cannot satisfy the specifications. For example, TPU is among the very few materials in 3D printing with rubber-like behavior, as already mentioned.

Finally, it should be noted that no further TGA investigation was required, for example, before the extrusion process of the raw materials. TGA was conducted on the samples. to ensure and verify that the temperatures used for the filament extrusion and on the nozzle of the 3D printing head during the 3D printing process were lower than the temperatures at which the polymers studied herein start to degrade due to the thermal loads. This was necessary since any possible degradation of the polymers studied during the extrusion or the 3D printing process would have affected the mechanical test results. The TGA showed that no such phenomena occurred during the processes followed in the study. The authors have conducted research in the past on polymers, such as PETG, studied herein [72], examining their performance during recycling and more specifically after six repetitions of a thermomechanical course, i.e., extrusion and 3D printing (six such sets). In this work, TGA was conducted after each thermomechanical course, and mechanical tests were conducted as well. It was found that the thermal reprocessing up to the six repetitions studied does not negatively affect the thermal stability or the mechanical properties of the polymer. On the contrary, the mechanical properties are improved up to the third repetition in the case of PETG. Similar behavior in the polymers when reprocessed has been reported by other researchers as well [73].

## 5. Conclusions

This study refers to the compression sensitivity analysis of four extensively used polymers in the FFF additive manufacturing process. For the fabrication of the analyzed specimens, the 3D printing parameters were set in such a way as to ensure the best quality with solid infill without porous or internal two- or three-dimensional imperfections. The calculation of the compression strain interprets the compressive behavior of all four polymers for various compression speeds. The key findings of the study can be summarized as follows:

- Due to the strain hardening effect, the compressive strength increased in all polymers with an increase in the test speed. Only in the PC polymer did the compressive strength increase up to the 150 mm/min test speed, and then it decreased at the highest test speed of 200 mm/min.
- The yield stress followed a similar pattern as the compression strength in all polymers tested herein.
- The PC and the PETG polymers had slightly different responses in the tests, justifying the need for individual experiments for each polymer.
- The calculation of the sensitivity index (“m”) and compressive toughness under different compression conditions can be used as valuable design parameters in future research works, indicating how a material is affected by changes in loading conditions.
- PC had the highest sensitivity index of the four polymers, and TPU was second, while PMMA and PETG had similar and significantly lower sensitivity index values.
- Differences were observed in the morphological characteristics of the four polymers at failure. The developed cracks differed, showing a different response of the polymers, with TPU not developing any cracks up to the highest test speed of 200 mm/min.

The polymers investigated here are widely used in a variety of applications in various industries. Detailed knowledge of the mechanical characteristics of these materials, especially under extreme conditions, is of paramount importance, especially in defense- and security-related applications. Having lightweight armor exhibiting high compressive strength and robustness in ballistic or blast loading is of key importance. It has been proven that it can save lives and reduce fatal or critical injuries for those using that kind of protective equipment either as a vest or as a helmet. In future work, research can be expanded to higher test speeds. The effect of the 3D printing parameters on the mechanical performance of the four polymers studied under various test speeds can be also evaluated.

## Figures and Tables

**Table 1:** Extrusion process (filament fabrication) settings for the four polymers.

	PC	PETG	PMMA	TPU
Heating Zone 1 (°C)	240	180	235	205
Heating Zone 2 (°C)	240	200	235	205
Heating Zone 3 (°C)	240	200	235	205
Heating Zone 4 (°C)	200	180	225	185
Rotation speed of screw (rpm)	4.8	5	11	9.7
Rotational speed of winder (rpm)	Automatic	Automatic	Automatic	Automatic

**Table 2:** The selected 3D printing settings for the fabrication of the specimens.

	PC	PETG	PMMA	TPU
Printing speed (mm/s)	40	40	40	40
Extrusion temperature (°C)	260	240	250	215
Bed temperature (°C)	85	70	110	60

## Data Availability

The data presented in this study are available on request from the corresponding author.
